# *Yersinia enterocolitica*: Mode of Transmission, Molecular Insights of Virulence, and Pathogenesis of Infection

**DOI:** 10.4061/2011/429069

**Published:** 2011-09-07

**Authors:** Yeasmin Sabina, Atiqur Rahman, Ramesh Chandra Ray, Didier Montet

**Affiliations:** ^1^Department of Genetic Engineering and Biotechnology, University of Dhaka, Dhaka 1000, Bangladesh; ^2^Department of Microbiology, University of Dhaka, Dhaka 1000, Bangladesh; ^3^Central Tuber Crops Research Institute, Bhubaneswar, India; ^4^Centre International de Recherche en Agronomie pour le Developpement (CIRAD), Montpellier, France

## Abstract

Although *Yersinia enterocolitica* is usually transmitted through contaminated food and untreated water, occasional transmission such as human-to-human, animal-to-human and blood transfusion associated transmission have also identified in human disease. Of the six *Y. enterocolitica* biotypes, the virulence of the pathogenic biotypes, namely, 1B and 2–5 is attributed to the presence of a highly conserved 70-kb virulence plasmid, termed pYV/pCD and certain chromosomal genes. Some biotype 1A strains, despite lacking virulence plasmid (pYV) and traditional chromosomal virulence genes, are isolated frequently from humans with gastrointestinal diseases similar to that produced by isolates belonging known pathogenic biotypes. *Y. enterocolitica* pathogenic biotypes have evolved two major properties: the ability to penetrate the intestinal wall, which is thought to be controlled by plasmid genes, and the production of heat-stable enterotoxin, which is controlled by chromosomal genes.

## 1. Introduction

*Yersinia enterocolitica* was discovered more than 60 years ago [1] but was not considered as a human or veterinary pathogen until the late 1960s when it became increasingly identified in foodborne gastrointestinal infections [2, 3]. *Y. enterocolitica* is a member of the genus *Yersinia* which encompasses a heterogeneous collection of facultatively anaerobic bacteria that belong to the family Enterobacteriaceae. Of the 11 species within this genus [4], only three, *Y. pestis, Y. pseudotuberculosis,* and *Y. enterocolitica* are regarded as pathogenic for humans whereas *Y. ruckeri* is a fish pathogen, and *Y. enterocolitica*-like organisms *Y. krirtensenii, Y. intermedia, Y. mollaretii, Y. frederiksenii* and *Y. bercovieri* have yet an unidentified role in human disease [5]. *Y. enterocolitica* is associated with a wide range of clinical and immunological manifestations, responsible for intestinal diseases, including enterocolitis with an inflammatory diarrhea in affected infants and young children; acute terminal ileitis and mesenteric lymphadenitis mimicking appendicitis in older children and young adults, as well as rare extraintestinal manifestations including urinary tract and respiratory tract infection (empyema), osteoarticular infection (reactive arthritis), erythema nodosum, infected mycotic aneurysm [6–8], axillary abscesses [9], and endocarditis [10]. 

The geographical distribution of *Y. enterocolitica *is diverse. *Y. enterocolitica *has more than 50 distinct serotypes (on the basis of antigenic variations in cell wall lipopolysaccharide), and few of them are pathogenic. O:8 is the primary infectious serotype in the USA followed by O:3, O:5, 27, O:13a,13b, O:20, O:9, and so forth [6, 7]. In China, serotype O:3 is primarily found in infections followed by O:9 and O:8 [14]. Furthermore, various serotypes demonstrate geographical specificity; for example, the predominant serotype in Australia, Europe, and Canada is O:3 [5], O:8 in Japan [15], and O:9 in Scandinavia, the Netherlands [16].

The incidence of *Y. enterocolitica *foodborne infection varies according to geography and climate variation. In developed countries, the incidence is higher in infants and young children, although all ages are at risk. The majority of foodborne infections are sporadic, and the infection sources are unknown, but large outbreaks have also occurred [5, 17, 18]. *Y. enterocolitica* foodborne outbreaks have occurred in Australia, Finland, Japan, Norway, the United States, and Brazil. There were two foodborne outbreaks in China in 1980s; one was caused by beef contamination in Lanzhou of Gansu Province in 1986 with 109 patients with diarrhea caused by *Y. enterocolitica* O:3 infection [19]. The second occurred in a school in Shenyang of Liaoning Province with 352 students having diarrhea caused by *Y. enterocolitica* O:8 infection [14]. Recently, *Y. enterocolitica* has become of concern worldwide, and foodborne infections have been reported in hundreds of countries. 

## 2. Mode of Transmission

### 2.1. Foodborne Transmission

*Y. enterocolitica *is an important foodborne human enteropathogen that causes sporadic illness and occasional foodborne outbreaks in the United States whereas incidence of yersiniosis and outbreaks appeared to be higher in many European countries than the United States [6, 20]. It has been isolated from many foods, including beef, pork, liquid eggs, soft cheese, raw milk, pasteurized milk, fish, raw oysters, shrimps, crabs, chocolate milk, turkey, chow mein (chop suey served with fried noodles), powdered milk, bean sprouts (especially mung beans, lentils, or edible soybeans), and tofu (cheese-like food made of curdled soybean milk). Although the organism has been isolated from many foods, there have been relatively few foodborne outbreaks attributed to *Y. enterocolitica *in developed countries, for example, Japan and the Netherlands [15, 16] as well as in developing countries, for example, Bangladesh and Iraq [21, 22]. Human yersiniosis is primarily acquired through the gastrointestinal tract as a result of ingestion of contaminated foods—usually raw or inadequately cooked pork [16]. *Y. enterocolitica* foodborne outbreaks in the United States have involved young children exposed indirectly during the cleaning and preparation of raw or undercooked pork chitterlings [23]. Chitterlings are generally well cooked, so it is believed that hands, kitchen surfaces, or other kitchen articles contaminated during the preparation of chitterlings are the vehicles for foodborne infection. Survival of *Yersinia* on these vehicles is facilitated by the hardiness of *Yersinia*, which is able to multiply in adverse conditions like commercial refrigeration temperatures. Other foodborne outbreaks have been associated with untreated water, contaminated tofu, contaminated bean sprouts, and contaminated milk (unpasteurized or inadequately pasteurized milk) [20]. The isolation of *Yersinia *strains from contaminated milk can be probably the result of postpasteurization contamination, since even the most heat-resistant strains are reported to be killed by pasteurization.

### 2.2. Human-to-Human Transmission

Person-to-person transmission is rare. However, contamination of food by infected food handler and nosocomial infections have been reported. In July 2006, person-to-person transmission was observed in a familial outbreak of *Y. enterocolitica* bioserotype 2/O:9 in Japan [24]. The possible source of this infection was an infected carrier who suffered from diarrhea [24]. In addition, the outbreak of diarrheal disease due to *Yersinia enterocolitica* bioserotype 1/0:5 was reported in hospitalized patients, which was the indication of a nosocomial outbreak due to *Yersinia enterocolitica *[25].

### 2.3. Animal-to-Human Transmission and Waterborne Transmission

Occasionally *Y. enterocolitica* infection occurs after direct or indirect contact with infected animals. It has been isolated from the intestinal tracts and feces of many animals, including rodents (rabbits), domestic animals (e.g., sheep, cattle, cats, pigs, and dogs) [26], and other animals (deer, raccoons, and horses) and water contaminated by those animals. The pig appears to be the main reservoir for the strains causing infection in humans. Pig feces are a potential mode of direct transmission to farmers [27]. As *Y. enterocolitica* possess the ability to grow under extreme environmental condition, they are welladapted to survival in cooler temperate zones as well as in microaerophilic environments including aquatic environments.

### 2.4. Direct Transmission

*Y. enterocolitica* rarely causes extraintestinal disease. In case of extraintestinal disease, direct transmission is proposed as the mode of transmission of this classically enteric pathogen [9]. In January 2009, a 54-year-old African American construction worker with chronic hepatitis C developed an axillary abscess due to *Y. enterocolitica* that followed an injury to his finger. It was proposed that the finger pustule arising as a consequence of traumatic puncture presented the possibility that direct inoculation of *Y. enterocolitica* from an environmental source may have been the mode of transmission. These suggest an alternative nonfoodborne route for *Y. enterocolitica* transmission. A similar route of transmission was proposed for a patient with *Y. enterocolitica* axillary abscess whose employment as a butcher subjected him to frequent cut wounds to the hand [28].

### 2.5. Blood Transfusion-Associated Transmission

*Yersinia enterocolitica* can be transmitted through contaminated blood, and it was one of the first recognized causes of posttransfusion sepsis [29]. This first case of transfusion-associated sepsis caused by *Y. enterocolitica* was described in the Netherlands in 1975. Since then, more than 60 additional cases have been reported in the literature worldwide. *Y. enterocolitica* has occurred occasionally in donor blood from healthy donors or donors with a diarrhea history; such contaminated blood sometimes caused *Yersinia* bacteremia and death of the recipients [30]. Although fatality due to posttransfusion bacterial-associated sepsis is rare [31], blood-transfusion-associated septicemia due to *Y. enterocolitica* is reported to have high fatality rate. In 2003, a fatal case of septic shock was observed in a 71-year-old patient following transfusion of contaminated red blood cells (RBCs) for refractory anemia. *Y. enterocolitica *bioserotype 4/O:3 was isolated from the patient's blood sample and the transfused RBCs. High titers of antibodies against *Y. enterocolitica* were detected in the donor's plasma sample one month after blood donation. The donor reported abdominal discomfort 3.5 months before blood collection but had no clinical signs of intestinal infection at the time of donation [32].

## 3. Molecular Insights in Virulence

*Yersinia enterocolitica* has evolved into an apparently heterogeneous collection of organisms encompassing six biotypes differentiated by physiochemical and biochemical tests (1A, 1B, 2, 3, 4, and 5; Table 1) and more than 50 serotypes differentiated by antigenic variation in cell wall lipopolysaccharide. Of the six biotypes, biotype 1A is the most heterogeneous, and encompasses a wide range of serotypes (Table 2), of which serotypes O:5, O:6,30, O:6,31, O:7,8, O:10, as well as O-nontypable strains, are isolated most often [33]. The virulence of the pathogenic biotypes, namely, 1B and 2–5 is attributed to the presence of a highly conserved 70-kb virulence plasmid, termed pYV/pCD and certain chromosomal genes [42] (Table 3). The biotype 1A strains of *Y. enterocolitica*, on the other hand, have been reported to lack pYV plasmid which encodes virulence factors including *Yersinia* adhesin A (YadA) and Ysc-Yop type III secretion system (TTSS) as well as chromosomally borne virulence genes including *ail, myfA, ystA, ysa*, and the high pathogenicity island- (HPI-) associated iron acquisition system [35]. 

### 3.1. Virulence Factors of pYV-Bearing Strains of *Y. enterocolitica* [33]

Apart from pYV itself, pYV-bearing strains of *Y. enterocolitica* require a number of chromosomally borne genes to express full virulence. Some of these virulence genes are restricted to pYV-bearing bacteria whereas others occur more widely. Virulence genes that are mostly limited to pYV-bearing strains of *Y. enterocolitica* include *inv* (encodes invasin, an outer membrane protein that is required for efficient translocation of bacteria across the intestinal epithelium) [43]); *ail* (encodes another outer membrane protein that may contribute to adhesion, invasion, and resistance to complement-mediated lysis) [44]; *yst *(encodes *Yersinia* stable heat-stable enterotoxin that may contribute to the pathogenesis of diarrhea associated with acute yersiniosis) [45, 46]; *myf* (encodes a fimbrial antigen and putative adhesin) [47]. In addition, strains of biotype 1B, which are particularly virulent for humans and laboratory animals, carry a high-pathogenicity island (HPI) which facilitates the uptake and utilization of iron by bacterial cells, and hence may promote their growth under iron-limiting conditions in host tissues [48]. Virulence-associated determinants of pYV-bearing *Y. enterocolitica* that also occur in pYV-negative strains include cell surface lipopolysaccharide and SodA (a superoxide dismutase), which appear to facilitate bacterial survival in tissues [49, 50], as well as urease, which enhances bacterial resistance to stomach acid and may also play a role in nitrogen assimilation [51].

pYV functions mainly as an antihost plasmid that permits the bacteria which carry it to resist to phagocytosis and complement-mediated lysis, thus allowing them to proliferate extracellularly in tissues. The pYV plasmid-encoded virulence factors include an outer membrane protein adhesin, YadA, and a type III protein secretory apparatus which translocates effector proteins, known as Ysc-Yops, from the bacterial cell to the cytoplasm of susceptible host cells [42]. The contribution of pYV-encoded factors, in particular YadA and the Yop effectors, to bacterial virulence has been established in a large number of studies. Strains of *Yersinia* which lack pYV are susceptible to killing by complement and polymorphonuclear leukocytes, although they are able to persist in macrophages and nonprofessional phagocytic cells, and cause short-lived infections which are typically asymptomatic [52].

### 3.2. Evidence Indicating the Lack of Virulence of Biotype 1A Strains

Biotype 1A strains of *Y. enterocolitica* are often considered to be nonpathogenic primarily because they do not possess the virulence-associated factors of pYV-bearing strains. The biotype 1A strains have been reported to lack both pYV plasmid and most chromosomal virulence genes such as *ail, myfA, ystA, ysa,* TTSS, and HPI, and only occasionally carry *ystA *and* myfA* [53]. Although the *ail *gene is present in some biotype 1A strains, the *ail* gene alone is an insufficient virulence marker for detecting the virulence of *Y. enterocolitica *biotype 1A strains [54]. Another line of evidence that is taken to indicate the avirulence of biotype 1A strains is their relatively high prevalence in the environment and healthy animals. Indeed, biotype 1A strains are ubiquitous, inhabiting a wide variety of environmental niches such as soil and various sources of water, including streams, lakes, water wells, and wastewater [55, 56] Sharon et al. 2003. They are also frequently isolated from foods, including various vegetables and animal products, such as pork, poultry, packaged meat, seafood, raw milk, and pasteurized dairy products. Biotype1A are also found in a vast array of animals, including birds, fish, various insects, frogs, and a wide range of mammals, including cattle, sheep, pigs, and rodents. In most cases, animals infected with biotype 1A strains are asymptomatic, thus giving support to the concept that these bacteria are avirulent commensals [33] (Table 4).

### 3.3. Some Studies Indicating the Pathogenicity in Some *Y. enterocolitica* Biotype 1A Strains

Despite the lack of traditional chromosomal-borne and plasmid-borne virulence genes in *Y. enterocolitica* strains of biotype 1A, some biotype 1A strains are isolated frequently from humans with gastrointestinal diseases. The biotype 1A strains isolated from humans and from pigs have been reported to produce *ystB-encoding Yersinia* heat-stable enterotoxin [53]. A recent study on 259 isolates of *Y. enterocolitica* and related species; indicated that Yst-B (*ystB*) was the major contributor to diarrhea produced by biotype 1A strains of *Y. enterocolitica* [62]. Some biotype 1A strains produce symptoms indistinguishable from that produced by isolates belonging to pathogenic biotypes [63, 64]. Biotype 1A strains have also been implicated in nosocomial [25] and foodborne [65] outbreaks, and were also isolated from extraintestinal infections [66].

## 4. Pathogenesis

*Yersinia enterocolitica* pathogenesis is incompletely understood. Most isolates of *Y. enterocolitica* from food or clinical materials have either of two pathogenic properties. First property is the ability to penetrate the intestinal wall, which is thought to be controlled by 70-kb virulence plasmid (pYV/pCD) genes; that is absent in avirulent strains; second one is the production of heat-stable enterotoxin which is controlled by chromosomal genes (*ystA, ystB,* and *ystC*) [61].

### 4.1. Adaptation

As contaminated foods are considered as the common mode of transmission, this microorganism must first adapt its surface antigenic structures like outer membrane proteins to colonize in the intestines of humans at a temperature of about 37°C. This is usually achieved in part through the presence of 70-kb virulence plasmid (pYV). Genes on this plasmid encode for several outer membrane proteins (polypeptides) that are expressed at 37°C but not at 25°C [6].

### 4.2. Adhesion

Attachment of pYV-bearing strains (pathogenic biotypes 1B and 2–5) of *Y. enterocolitica* to tissue culture cells like HeLa cells or HEp-2 cells cultures has been frequently identified in pathogenic *Yersinia* isolates [5, 67, 68]. However, the ability to produce disease does not correlate with HeLa cell attachment as plasmid cured avirulent strains retain the ability to attach to HeLa cells [69]. When the pYV plasmid-containing strain was grown at 26°C in calcium-containing medium, the bacteria adhered to HeLa cells and HEp-2 cell cultures to a high degree. In contrast, when this strain was incubated at 37°C in the same calcium-containing medium, it attached to the HeLa cells and HEp-2 cell cultures at a reduced level [70]. By insertional inactivation of genes located on the virulence plasmid (pYV), Kapperud et al. [71] identified four plasmid-dependent and temperature-inducible properties related to the bacterial surface properties involved in fimbrial adhesion: (i) a fimbrial matrix covering the outer membrane, (ii) outer membrane protein, YOP1 which is a structural component of the fimbriae, (iii) spontaneous autoagglutination, which is related to the fimbriae, and (iv) mannose-resistant hemagglutination of guinea pig erythrocytes [71]. 

Although the biotype 1A strains of *Y. enterocolitica* have been reported to lack pYV plasmid, various forms of fimbriae are observed in this biotype. One of fimbriae, designated MR/Y-HA is 8 nm in diameter, agglutinates erythrocytes of 10 different animal species in the presence of mannose and is expressed in vitro at low temperature, but not at 37°C [72]. A second type of fimbriae, designated MR/K-like HA, is 4 nm in diameter and mediates mannose-resistant hemagglutination of chicken erythrocytes, but not erythrocytes from a variety of other species [72]. Expression of these fimbriae in vitro occurs only after serial passages of bacteria for at least 7 days. Moreover, as they do not mediate adherence of bacteria to cultured epithelial cells [73], their contribution to the pathogenesis of infection with biotype 1A strains is unknown [33].

Some strains of *Y. enterocolitica* produce a fimbrial adhesin, named Myf (for mucoid *Yersinia *fibrillae), because it bestows a mucoid appearance on bacterial colonies which express it. Myf are narrow flexible fimbriae which resemble CS3, an essential colonization factor of some human clinical strains of enterotoxigenic *Escherichia coli* [33]. However, *myf *genes-associated virulence of these bacteria is unknown.

### 4.3. Invasiveness (Mechanisms of Epithelial Cell Invasion)

Entry of enteroinvasive bacteria into the intestinal epithelial cell is the key to a successful invasive process. The ability of *Y. enterocolitica* to invade epithelial cells is an important correlation of pathogenicity [8]. The invasive process includes a major signalling process that an invasive microorganism may provoke to force its way into a nonphagocytic cell, and then disrupting and invading the intestinal barrier, a process that involves interaction with other cellular components of this barrier. There are essentially two major mechanisms of bacterial epithelial cell internalization [74] The “zippering” process corresponds to tight enclosing of the bacterial cell by the mammalian cell membrane, involving a surface bound bacterial protein binding an adherence molecule of the mammalian cell surface with high affinity—that is, the invasin (Inv) of *Yersinia *binding integrins of the *β*1 family of mammalian cell surface [75]. One reason that strains of biotype 1A have been considered to be avirulent is that they invade tissue culture cells to a lesser extent than pYV-bearing strains [69, 76]. However, paradoxically, some pYV-bearing strains themselves may retard mammalian epithelial cell invasion via the effects of translocated Yops on cytoskeletal proteins [42] as well as some biotype 1A strains are positive for the *ail *gene encoded an outer membrane protein that may contribute to epithelial cell adhesion and invasion [77].

### 4.4. Local and Systemic Dissemination

**Figure 4:**
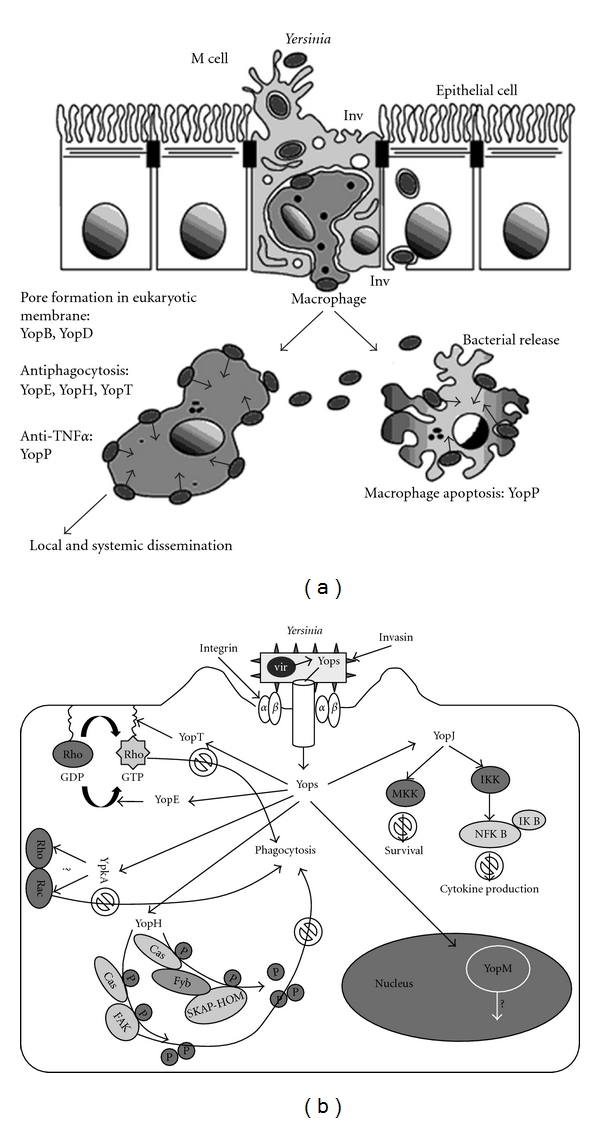
Physiopathological scheme of *Yersinia* infection (adapted from [13]). The Yops are delivered into the host cells via a type III secretion system. YopH, a tyrosine phosphatase, dephosphorylates Cas and FAK (protein tyrosine kinase) in epithelial cells, and Cas, Fyb, and SKAP-HOM in macrophages that are involved in the assembly of cytoskeletal complexes required for phagocytosis [78]; YopT modifies the Rho family GTPases by inducing redistribution of the RhoA GTPase [79]; YopE inactivates the Rho family of GTPases involved in phagocytosis [80]; YpkA binds to Rac and Rho (function unknown). These four Yops alter or disrupt the actin cytoskeleton and thereby block phagocytosis. YopJ impairs activation of MAPKKs and NF-B, which induces apoptosis and inhibits cytokine production. YopM is translocated into the nucleus (function unknown).

*Y. enterocolitica* usually causes a diarrheal disease, and sometimes systemic diffusion. *Yersinia* virulent strains cross the intestinal epithelium primarily through the FAE (follicle associated epithelial cell), in the Peyer's patches of the ileum [81]. Invasin (Inv), a 103 kDa outer membrane protein of *Yersinia* binds *β*1 integrins that are also expressed apically on M cells. Inv negative mutants still adhere to and invade M cells, but at a much lower level than the wildtype strain and their colonization potential for Peyer's patches is considerably reduced [82]. Other *Yersinia *surface proteins such as Ail, PsaA, and YadA may account for residual invasion of *inv* mutants [83]. After invasion process, *Yersinia* defend the attack by resident macrophages by expressing an antiphagocytic strategy mediated by a plasmid encoded type III secretion, of three protein effectors, YopH, T, and E, that disrupt cytoskeletal assembly required for phagocytosis process [84, 85]. *Yersinia* strains therefore remain extracellular in infected Peyer's patches and mesenteric lymph nodes, and then disseminate to cause local and systemic infection (Figure 4).

## 5. Conclusion

*Yersinia enterocolitica* is most often transmitted by consumption of contaminated food (most commonly raw or undercooked pork), unpasteurized milk or inadequately pasteurized milk, untreated water, or by direct or indirect contact with animals. The virulence of *Y. enterocolitica* strains mostly depends on the presence of pYV plasmid. *Y. enterocolitica *pYV-positive strains contain plasmid-mediated virulence genes involved in developing infection especially in gastrointestinal tract with the help of traditional chromosomal genes whereas pYV-negative strains are mostly noninfectious except heat-stable enterotoxin-producing strains.

## Figures and Tables

**Figure 1:**
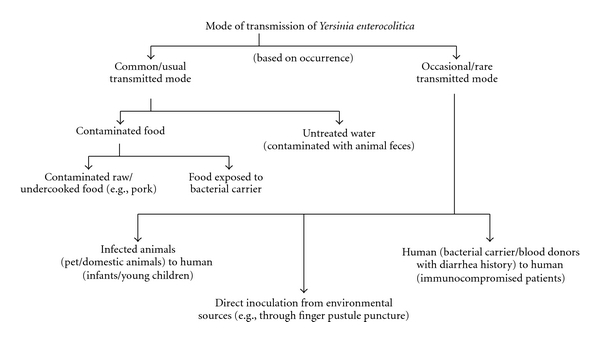
Mode of transmission of *Y. enterocolitica. *

**Figure 2:**
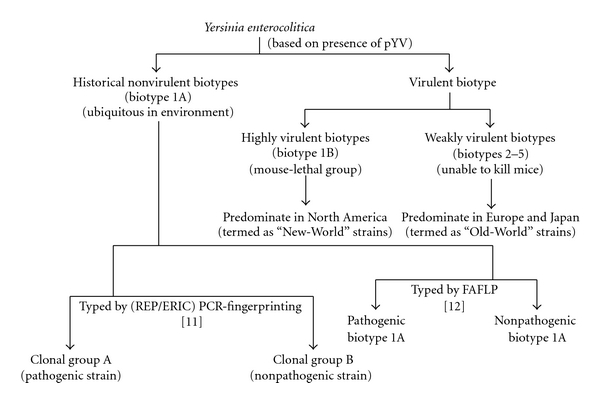
*Y. enterocolitica *biotypes. *Y. enterocolitica *biotypes are classified into three distinct group: a historically defined nonpathogenic group (biogroup 1A); a weakly pathogenic group that are unable to kill mice (biogroups 2 to 5); a highly pathogenic, mouse-lethal group (biogroup 1B). Biotype 1A strains are clustered into two clonal groups (A and B) when typed by repetitive extragenic palindrome (REP)—and enterobacterial repetitive intergenic consensus (ERIC)—PCR fngerprinting [11], and two groups when typed by fluorescent amplified fragment length polymorphism (FAFLP) [12].

**Figure 3:**
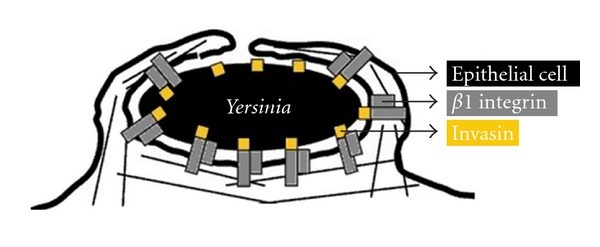
A paradigm of “zippering” entry of a bacterial pathogen into epithelial cells. Invasin mediated binding of Yersinia to *β*1 integrins and internalization (adapted from [13]).

**Table 1:** Biotyping scheme of *Y. enterocolitica* (adapted from [33, 34]).

Test	Reaction of biotype					
	1A	1B	2	3	4	5
Lipase (Tween hydrolysis)	+	+	−	−	−	−
Aesculin hydrolysis	V	−	−	−	−	−
Indole production	+	+	(+)	−	−	−
D-Xylose fermentation	+	+	+	+	−	v
Voges-Proskauer reaction	+	+	+	+	+	(+)
Trehalose fermentation	+	+	+	+	+	−
Nitrate reduction	+	+	+	+	+	−
Pyrazinamidase	+	−	−	−	−	−
B-D-Glucosidase	+	−	−	−	−	−
Proline peptidase	v	−	−	−	−	−

+, positive; (+), delayed positive; –, negative; v, variable reactions.

**Table 2:** Relationship between biotype, O serotype, and pYV carriage of *Y. enterocolitica *(adapted from [33]).

Biotype	Serotype(s)
1A	O:4; O:5; O:6,30; O6,31; O:7,8; O:7,13; O:10; O:14; O:16; O:21; O:22; O:25; O:37; O:41,42; O:46; O:47; O:57; NT^a^
1B	O:4,32^b^; O:8^b^; O:13a,13b^b^; O:16; O:18^b^; O:20^b^; O:21^b^; O:25; O:41,42; NT
2	O:5,27^b^; O:9^b^; O:27
3	O:1,2,3^b^; O:3^b^; O:5,27^b^
4	O:3^b^
5	O:2,3^b^

^a^NT, not typable.

^b^Serotypes which include strains that carry pYV.

**Table 3:** Virulence-associated genes in *Y. enterocolitica. *

Genes	Gene product/function	Reference
*Inv^C,tr^*	Invasin (an outer membrane protein that is required for efficient translocation of bacteria across the intestinal epithelium)	[35]
*ail^C,tr^*	Adhesin (outer membrane protein that may contribute to adhesion, invasion, and resistance to complement-mediated lysis)	[36]
*virF^P,tr^*	Transcriptional activator	[36]
*myfA^C^*	Mucoid *Yersinia* factor (fimbrial antigen and putative adhesin)	[37]
*ystA^C,tr^*	Enterotoxin (*Yersinia* stable heat-stable toxin that may contribute to the pathogenesis of diarrhea)	[38]
*ystB^C^*	Enterotoxin (*Yersinia* stable heat-stable toxin that may contribute to the pathogenesis of diarrhea)	[38]
*ystC^C^*	Enterotoxin (*Yersinia* stable heat-stable toxin that may contribute to the pathogenesis of diarrhea)	[39]
*fepA*	Enterochelin receptor protein	[40]
*fedD*	Enterochelin receptor protein	[40]
*Fes*	Enterochelin esterase	[40]
*tccC^P^*	Insecticidal toxin-like protease	[35]
*ymoA*	*Yersinia* modulator	[41]
*hreP*	Subtilisin/kexin-like protease (host responsive element)	[35]
*Sat*	Streptogramin acetyltranferase	[35]
*yadA^P,tr^*	*Yersinia* adhesin A	[42]
*ysa^P^*	*Yesinia* secretion apparatus	[42]

*^C^Chromosome borne gene, ^P^plasmid-borne gene, ^tr^traditional virulence gene.*

**Table 4:** Studies indicating the lack of virulence of biotype 1A strains.

Research studies	References
(1) Two large studies in Belgium, involving the microbiological investigation of more than 24,000 fecal samples over a period of almost 16 years, revealed that infection with biotype 1A was not associated with gastrointestinal symptoms and that biotype 1A strains were more frequent amongst subjects having no gastrointestinal complaints.	Van Noyen et al. [57, 58]
(2) Rabbits were infected perorally with different biotype 1A strains from raw fish (serotype O:6,30) and pig intestine (serotype O:5), respectively, and concluded that these bacteria were avirulent.	Pai et al. [59]Une [60]
(3) Robins-Browne et al. reported that gnotobiotic piglets, inoculated perorally with a biotype 1A strain of serotype O:5, which was originally isolated from milk, rapidly cleared the bacteria without developing any clinical or pathological evidence of disease.	Robins-Browne et al. [61]
